# How Can E-Cigarette Fear Appeals Improve the Perceived Threat, Fear, Anger, and Protection Motivation of Young People

**DOI:** 10.3389/fpsyg.2021.676363

**Published:** 2021-08-30

**Authors:** Chengzhi Sun, Fangfei Wang, Mengmeng Jiang

**Affiliations:** ^1^School of Foreign Languages, Dalian University of Technology, Dalian, China; ^2^Faculty of Humanities and Social Sciences, Dalian University of Technology (DUT), Dalian, China

**Keywords:** Extended Parallel Process Model, e-cigarettes, fear appeals, Appraisal-Tendency Framework, emotions

## Abstract

The lack of awareness regarding the risks of e-cigarettes and the misleading business propaganda caused an increase in the popularity of e-cigarettes among young people. The effective communication of the risks associated with e-cigarettes is an important part of current work to control their usage, and the use of fear appeals is an effective method to achieve good control. Based on the Extended Parallel Process Model (EPPM) and Appraisal-Tendency Framework (ATF), this article presents a 2 × 2 control experiment to test the impact of fear appeals on the perception of risk, emotions, and behavioral motivation of young people aged 35 and less. A total of 333 valid samples of adolescents and young adults were included to investigate the different response paths to fear appeals among young people of different age, sex and smoking history. The results show that high-threat, high-efficacy fear appeals are able to: (1) significantly increase young people’s perception of the e-cigarette-associated threats, (2) trigger fear and anger amongst young people, and (3) stimulate their self-protection motivation. Fear appeals do not have an impact on young people’s perception of efficacy, regardless of their level of threat and efficacy. High fear appeals can also increase young people’s perception of threat, which in turn enhances their anger and protection motivation. Furthermore, while this type of fear appeal can enhance young women’s perception of efficacy, it cannot enhance the perception of e-cigarette risks in adolescents, young men and young smokers, regardless of their level of threat and efficacy. Young non-smokers have a higher perception of the risks involved in the use of e-cigarettes compared with young smokers.

## Introduction

E-cigarettes are claimed to be mainly “tar-free, low-nicotine” and “helpful for smoking cessation” in China’s previous advertisements (Zhang et al., 2019), thus making 71% of Chinese young people believe that e-cigarettes are safer than traditional cigarettes (People’s Daily Online, 2019). However, many recent studies have found that despite the fact that they do not contain nicotine, e-cigarettes can also pose risks for both users and non-users through second-hand emissions, just like traditional cigarettes. The short-term or long-term use of e-cigarettes can have harmful effects on the individual’s respiratory system, cardiovascular system, nervous system, digestive system, and others (Meo and Al Asiri, 2014; Layden et al., 2019). However, due to the convenience of online shopping, misleading business propaganda and the lack of awareness regarding the risks associated with e-cigarettes, the total number of e-cigarette users in China has reached 10 million, mainly consisting of young people (Chinese Center for Disease Control and Prevention, 2019). Given these facts, the public awareness of the risks associated with e-cigarettes needs to be increased in order to control and reduce the harmful effects of e-cigarettes on young people.

Combining academic research on health risk communication and the practical experience of tobacco control, the fear appeal theory is recognized by many scholars to be an effective strategy for persuasion and behavior intervention (Ruiter et al., 2014). In previous studies, researchers provided the public with an appropriate fear appeal threshold and clear and feasible risk aversion strategies to encourage them to adopt corresponding protective behaviors (Witte et al., 2001); thus, the focus was on the public’s reactions to fear appeals (Zhang, 2021). However, the focus in current research on fear appeal theory is more directed toward the public’s rational cognitive mechanism for information, and the emotional response created by fear appeals is still limited to “fear” (So, 2013). In China, only few empirical studies have been performed on e-cigarettes, particularly in different age groups, which limits the pertinence and validity of research conclusion. Therefore, this article integrates the Extended Parallel Process Model (EPPM) with the Appraisal-Tendency Framework (ATF). We systematically examine young people’s responses and emotional perceptions of fear appeals that are associated with e-cigarettes, and then explore the impact of these fear appeals on their behavior. This article aims to expand the theoretical perspective of fear appeals and provide suggestions to control the use of e-cigarette.

## Literature Review

### Fear Appeal Theory

The fear appeal theory is often applied by scholars when persuasive research is conducted on certain threatening or risky issues, such as health communication and environmental communication (Hoffland et al., 2015). By assuming that fear appeals of different intensities can produce different levels of information acceptance among people, studies have focused on investigating the public’s perception and evaluating fear appeals and their corresponding response mechanisms. Several theoretical perspectives have been proposed: (a) Fear Appeals Model (FAM), which is represented by the Inverted “*U*-Shaped” model and used to explore the conditions under which fear appeals can function (Janis, 1967); (b) The Parallel Process Model (PRM), which considers the different response modes adopted by the public to fear appeals and divides the resulting effects into the two different directions of “fear control process” and “danger control process” (Leventhal, 1970); (c) Protection Motivation Theory (PMT), which describes the mechanism of a fear appeal from the perspective of the public’s cognition (Rogers, 1975).

The fear appeal theory has been enriched and expanded in recent years. By integrating previous studies, Witte brought the “fear” variable back into the fear appeal theory and proposed the “Extended Parallel Process Model” (EPPM). This model indicates that the public’s assessment of fear appeals ranges from “perceived threat” (such as severity and susceptibility) to “perceived efficacy” (such as self-efficacy and response efficacy). Furthermore, three modes of action were identified: “no response,” “danger control process” (make adaptive changes) and “fear control process” (such as refusing to accept fear appeals) (Witte et al., 2001). Witte (1992) also attempted to explain the EPPM model by integrating the public’s cognitive and emotional response mechanisms. He indicated that the “danger control” protective behavior can be regarded as a cognitive process, which is stimulated under high-efficacy conditions, while “fear control process” and “no response” are generally “emotional processes,” which are stimulated under low-efficacy conditions.

Meanwhile, the influence of emotions on public behavior has also been investigated to identify other types of public emotions and explore their pathways beyond the emotion of “fear.” Most studies have focused on two types of negative emotions, anxiety and anger. For example, some scholars have revealed that the public’s fear and anxiety, two emotions that are considered to be independent and discrete from a neurobiological point of view, were simultaneously aroused by fear appeals (Sylvers et al., 2011). However, the impact of anxiety can be more direct than that of fear on the public’s intentions to protect themselves (So et al., 2016). Anger has been shown by many studies to be significantly increased by fear appeals (Kim and Shin, 2017) and to have the effects of reducing the estimated risk level and risk prevention measures, as opposed to the effects of fear (Lerner et al., 2003).

### Appraisal-Tendency Framework

The Appraisal-Tendency Framework (ATF) was developed from the Cognitive Appraisal Theory and the Functional Theory of Emotion (Lerner and Keltner, 2000). Basically, this framework maintains the “appraisal tendency”: each emotion will activate a certain cognitive tendency according to the core appraisal dimension that has triggered this emotion, and related events are accordingly evaluated. The appraisal tendency is consistent with the original cognitive appraisal dimension of the emotion. In short, the appraisal process influences an individual’s cognition through emotion, thereby affecting their judgment and decision-making process, and finally solving the event that triggered the emotion (Lerner and Keltner, 2000). Different emotions can be distinguished according to their different core appraisal dimensions, which can be considered to determine the types of emotions that individuals generate. These emotions have an influence on the individual’s cognition and ultimately have an impact on the decision-making process of related events. However, the premise of this appraisal process is that the core appraisal dimension of the emotion needs to match the outstanding attributes of the event (Han et al., 2007). For example, if the main attribute of an event is control, then the occurrence of the event will induce a variety of emotions in the individual, but only relevant emotions with a core appraisal dimension that is control will have an impact on the event.

The Appraisal-Tendency Framework is primarily used in the field of psychological research. Many studies have investigated the different ways in which emotions affect risk, consumer, medical, or other behavioral decisions through appraisal and adjustment mechanisms. In communication studies, this theory is primarily used to study how different emotions affect different response decisions during risk and crisis communication. For example, Jin et al. (2012) used an experiment to explore the impact of the public’s certainty and controllability on their emotional responses to a crisis and the corresponding coping strategy preferences. Their results showed that the negative emotions that people are most likely to develop in a crisis are anger, fear, anxiety, and sadness. By examining the different influence of these four public emotions with different levels of certainty and controllability on the crises, they showed anger, fear and sadness to be significantly increased in crises that match their attributes, while anxiety was not. At the same time, it was shown that in predictable but uncontrollable crises, the public prefers rational responses, while perceptual responses are preferred in unpredictable and uncontrollable crises (Jin, 2010). Further studies have shown that fearful groups produce low-certainty appraisals and experience uncertainty in the decision-making process, which results in risk averse behavior (Fuming et al., 2015). The ATF theory can test the influence of emotion on an event and improve the predictive level of individual coping strategies accordingly. Hence, this theory can better predict the individual risk preferences in the case of risk or crisis events, thereby ensuring that the relevant risk communication is more effective. Therefore, different groups may perceive the risks posed by e-cigarettes in different ways. As their emotions differ, their corresponding behavioral decisions also differ.

The current study attempts to refine the emotions generated by fear appeals and introduces two types of risk-related emotions, anxiety and anger, in addition to fear in a cohort of young people. ATF is integrated into the analysis of fear appeals for an in-depth examination of their cognition, generated emotions and related behavior. We propose the theoretical model shown in Supplementary Figure 1 and the following questions:

Q1: What are the influence paths of e-cigarette fear appeals on the behavioral decisions of young people?

Q2: What role do fear, anxiety, and anger play in e-cigarette fear appeals in young people? Are there differences in the effects created by different negative emotions?

### Differences in the Perception of Smoking Risks Among Young People

In recent years, more and more attention has been paid to risk communication related to smoking in young people (Kikut et al., 2020), which also became the focus of official tobacco control research in China. Previous studies have revealed significant differences in the public’s awareness of cigarettes and smoking behavior (Anthony et al., 2008; Mu et al., 2020). Common factors included age, sex and whether the individuals are smokers or not (Moore et al., 2015; Piñeiro et al., 2016). Traditional non-smoking groups, such as teenagers and women, have also received more attention from the tobacco control organizations (Wagner et al., 2017; Perikleous et al., 2018).

Age has been shown by many studies to be an important factor in smoking behavior, particularly the age at which a person first starts smoking (Buchmann et al., 2013). A study in the United States has shown that most adult smokers are first exposed to tobacco during their adolescence (Sonya et al., 2012). In addition, several empirical studies have shown that individuals who start smoking earlier are more likely to become addicted (Breslau and Peterson, 1996) and have more difficulties quitting smoking (Chen and Millar, 1998). Hence, it is more effective to make them aware of the harmful effects of smoking at the earliest possible opportunity, than to try and persuade them to stop smoking once they have started, so it is vitally important that they receive tobacco control education. However, a 2019 tobacco survey of Chinese middle school students showed the need to improve the attitudes of middle school students toward smoking addiction, as only about 30% of these students believe it is difficult to quit smoking (Chinese Center for Disease Control and Prevention, 2020). Therefore, in order to develop future smoking control strategies for teenagers, the focus should be on the cognition, behavior and attitudes of teenagers toward smoking, the factors that predict or affect smoking behavior (such as the smoking behavior of a partner), whether or not their parents smoke, recognition of the harmful effects caused by second-hand smoking (Yan et al., 2017), tobacco advertisements, exposure to second-hand smoking, exposure to tobacco control communication (Lin et al., 2017), witnessing smoking behavior in movies (Sonya et al., 2012) and the teenagers’ attitudes toward anti-tobacco intervention measures (Lazard et al., 2018). Due to the increased popularity of e-cigarettes among young people in recent years, many corresponding studies have been carried out. Most of these studies compared the perception of risk between e-cigarettes and tobacco and their influencing factors (Leavens et al., 2021). For example, some studies have indicated that the non-smokers who have been exposed to e-cigarette advertisements perceive much lower smoking risks compared with those who have not been exposed to such advertisements. Therefore, Kim et al. (2019) suggested that e-cigarette advertisements should be regulated to minimize the exposure of teenagers to smoking. To this end, this paper will focus on exploring the different attitudes and emotions of young people and other age groups toward the use of e-cigarettes.

Although most smokers are men, tobacco companies have started to implement promotional activities to attract female consumers since the turn of the century, in an attempt to increase the number of female smokers (Johnson et al., 2016). Men and women are different in terms of their physiology, psychology and thinking modes; thus, their smoking behavior, preferences and perception of the risks associated with smoking also differ, and different risk communication strategies for e-cigarettes need to be adopted for both groups. For example, Pang found that women are more sensitive to the smell of combustible tobacco and e-cigarettes with fruit and mint flavoring are more attractive for them than tobacco. Therefore, sex-based differences can be observed in the effect of regulatory policies on the taste of e-cigarettes (Pang et al., 2020). An analysis of smoking-related posts on the anonymous social networking site Tumblr showed that 77% of the posts were published by female users. Compared with women, men prefer to publish pictures of hookah and alcohol. This information can be used to assist in the future development of more sex-specific tobacco control measures (Primack et al., 2016).

In addition, being a smoker has an important influence on the individual’s perception of the smoking-associated risks (Pancani and Rusconi, 2018). Some studies have focused only on smokers, while others have compared smokers with non-smokers. For example, 242 smokers and 241 non-smokers aged between 18 and 29 years were studied in South Korea by conducting a fear appeal experiment on the picture or text warnings that appear on cigarette packaging. The results of this study indicated that the picture warnings can increase a smoker’s motivation to stop smoking and a non-smoker’s intention not to start smoking. Smokers with strong self-efficacy and self-esteem were more likely to quit smoking. However, for non-smokers, the picture warnings only had an effect on the individuals with a strong self-efficacy, while no effect was observed on the individuals with a strong or weak self-esteem. Thus, this study indicated that the warnings carried on cigarette packaging have an important effect on young people in South Korea, but the effect differs between smokers and non-smokers (Chun et al., 2018).

Therefore, based on previous research, the current study will investigate a cohort of young people from the perspectives of age, sex and smoking status, to compare the impact of e-cigarette fear appeals on different subgroups. The following research question is thus proposed:

Q3: In terms of age, sex and smoker status, what differences exist in the response paths of young people to e-cigarette fear appeals?

## Materials and Methods

### Experiment Material

Since “threat” and “efficacy” are the core characteristics of a fear appeal according to the fear appeal theory, many scholars have designed the communication information around these two characteristics (Carcioppolo et al., 2013). A threat is the external stimulus brought about by the content of the information (Witte, 1991). In order to enhance the public awareness of the severity and susceptibility of the threat/risk, scholars often provide the public with appalling language or bloody pictures to depict the consequences of certain threats or risks (Rogers, 1975). Efficacy involves the public’s appraisal of the effectiveness and self-efficacy of the recommendations provided by the communicator (Rogers, 1975).

To explore the influence of e-cigarette fear appeals on the protective behavior of young people, we designed a 2 (high/low threat) × 2 (high/low efficacy) online experiment. We referred to both the Chinese official tobacco control propaganda materials and related literature to set four experimental fear appeal situations. The former was obtained from official Chinese channels, including the Chinese Center for Disease Control and Prevention and Chinese official media, such as People’s Daily and Xinhua News Agency, aiming to enhance the accuracy of the information. The latter was used to design specific expressions that can distinguish between different levels of threat and efficacy. According to literature, the level of threat in fear appeals can be distinguished by the application of specific data, adjectives and scary pictures (Chun et al., 2018; Zhang and Zhou, 2019), while the level of efficacy can be differentiated according to whether it provides easy and detailed preventative methods and the effectiveness of the recommendations (Cho and Charles, 2006). Before performing the formal online experiment, a small-scale preliminary survey was conducted and experimental material was modified according to the feedback from the interviewees. The specific experimental material that was used in the current study is shown in Supplementary Table 1.

### Measurements

#### Perceived Threat and Perceived Efficacy

Perceived threat and perceived efficacy involve young people’s appraisal of the characteristics of fear appeals and form the starting point for their subsequent emotional and behavioral motivation. In order to measure perceived threat and perceived efficacy, we adopted Witte’s (1996) Risk Behavior Diagnosis Scale (RBD), while integrating the Chinese context and knowledge of e-cigarettes by making suitable adjustments.

The measurement of perceived threat includes both the severity and susceptibility of the threat (Witte, 1992). The severity of the threat (Cronbach’s α = 0.910) involves three items (e.g., “Long-term use of e-cigarettes will lead to addiction”), measured on a 5-point Likert scale (1 = strongly disagree, 5 = strongly agree). The susceptibility of the threat is directly measured by a 7-point Likert scale question: ‘What is the probability that you will be harmed by e-cigarettes?’ (1 = impossible, 7 = very likely).

The measurement of perceived efficacy includes two aspects: self-efficacy and the effectiveness of the advice (Witte, 1992). Self-efficacy (Cronbach’s α = 0.901) comprises three items (e.g., “I have the ability to follow the recommendations in the material to prevent the harmful effects of e-cigarettes”). The effectiveness of the advice (Cronbach’s α = 0.879) is composed of two items (e.g., “The advice to prevent the harmful effects of e-cigarettes provided in this information is specific and effective”). All the five above-mentioned items are measured on a 5-point Likert scale (1 = strongly disagree, 5 = strongly agree).

#### Fear, Anxiety, and Anger

By considering different Chinese contexts, this study asks the direct question “How much has your fear/anxiety/anger intensified after reading the material?” Then, the answers are measured on a 7-point Likert scale (1 = no feelings, 7 = very strong feelings). In order to improve the accuracy of emotion measurement, participants were asked to look at a beautiful picture to relax before reading the experimental material.

#### Behavioral Motivation

In EPPM, there are two results of fear appeals: protection motivation and defense motivation. In this study, six items measured on a 5-point Likert-type scale (1 = strongly disagree, 5 = strongly agree) were used to measure the two types of behavioral motivation (Protection motivation: e.g., “I have never used e-cigarettes and I don’t want/will not use them,” Cronbach’s α = 0.844; defense motivation: e.g., “I don’t think e-cigarettes have the above hazards,” Cronbach’s α = 0.865). Since the attributes of these two variables are mutually exclusive (Witte, 1992), this can be used to check the quality of the questionnaire. Therefore, to facilitate the analysis, this study combined defense motivation and protection motivation into a single variable called “behavioral motivation,” which specifically refers to protection behavioral motivation.

#### Control Variables

Previous studies have indicated that individual characteristics are important factors influencing the cognition toward cigarettes (Anthony et al., 2008; Mu et al., 2020). This study used the following factors as control variables: educational experience (from “junior high school and below” to “Master’s level and above” in ascending order of 1–5), marital status (1 = married, 2 = unmarried, 3 = divorced or widowed), location (divided by administrative area, from “municipalities” to “other,” in ascending order of 1–6), income (an individual’s monthly income, from “no income” to “more than 20,000 ¥,” arranged in ascending order of 1–6) and personal risk knowledge level (a weighted average of the following two item scores: “Most e-cigarettes contain different levels of nicotine, which are equally addictive” and “E-cigarette smoke can irritate the respiratory tract and may cause heavy metal poisoning, cancer, cardiovascular disease, lung disease, and many other diseases”).

In addition, it has previously been confirmed that whether an individual is a smoker has an important influence on their perception of the smoking-associated risks (Chun et al., 2018). Therefore, this study also used smoking experience (1 = smoked, 2 = not smoked), and e-cigarette use experience (1 = used, 2 = aware of but have not used) as control variables.

### Participants

This investigation was carried out as a network control experiment. The questionnaire was distributed on the platform website of Wenjuanxing. A professional research company recruited the participants. The sex quota was established based on the number of e-cigarette consumers reported by the Chinese Centre for Disease Control and Prevention and CBNData, which is a Chinese data survey company known for big data analysis of online consumption (CBNData, 2019; Chinese Center for Disease Control and Prevention, 2019); thus, 70% of the participants were males.

In the questionnaire, the participants were randomly assigned by the system to read one of four experimental situations (2 × 2). Then, the backend of the questionnaire recorded the time the participants took to finish reading, and each participant was asked to answer questions about core information mentioned in the material to control the validity of the sample. Afterward, the participants were asked about perceived threat and perceived efficacy, fear, anxiety, anger, and behavioral motivation.

We eliminated invalid samples with a reading time less than 240 s, because this indicated that the participant was unable to correctly understand the core information provided in the material, and selected young people aged 35 and below. As a result, 333 valid samples were obtained. The distribution of the total samples and representative groups of the samples in each experimental situation are shown in Supplementary Table 2. Using the “pwr” package in the R software, statistical power analysis was conducted on our sample size with α err. Prob. = 0.05, power (1-β err. Prob.) = 0.8; the resulting detectable effect was of an approximately medium range (Cohen, 1988) with a Cohen’s *f* = 0.25.

## Findings

### High Fear Appeals Affect Three Emotions and Behavioral Motivations Through Perceived Threats

#### High Fear Appeals Are More Successful When Communicating Risks to Young People

In this study, the Stata SE 15.1 software was used to conduct path analysis based on the hypothetical model. The perceived threat, perceived efficacy, fear, anxiety, anger, and behavioral motivation of young people were in turn analyzed as dependent variables. The results of the path analysis are shown in Supplementary Figure 2. In Supplementary Figure 2, the hypothetical model is partially established, where solid lines represent established paths and dashed lines represent untenable paths. All the established paths are marked with the effect value (β) (^∗^ *p* < 0.05, ^∗∗^ *p* < 0.01, ^∗∗∗^ *p* < 0.001).

On the path of perceived threat, among the four groups of fear appeals, only a high-threat, high-efficacy fear appeal significantly improved the perceived threat of e-cigarettes experienced by young people (*p* = 0.045 < 0.05, *t* = 2.01, β = 0.134). Then, the pathway model for analyzing the effect of perceived threat on emotions and behavioral motivation was established, and mediation analysis was conducted to calculate the direct and indirect effects, as shown in Supplementary Figure 2. The results showed that perceived threat significantly increased the emotions of fear (*t* = 4.14, *p* = 0.000 < 0.001, *a*1 = 0.223) and anger (*t* = 3.19, *p* = 0.002 < 0.01, *a*3 = 0.177), but did not affect anxiety (*t* = 1.73, *p* = 0.095 > 0.05, *a*2 = 0.956). After controlling for the perceived threat, fear (*t* = 2.27, *p* = 0.024 < 0.05, *b*1 = 0.117), anxiety (*t* = 5.61, *p* = 0.000 < 0.001, *b*2 = 0.283) and anger (*t* = 3.83, *p* = 0.000 < 0.001, *b*3 = 0.192) were all significantly related to the behavioral motivation of young people. Furthermore, perceived threat was significantly and indirectly associated with the behavioral motivation through fear (*a*1*b*1 = 0.026, *p* = 0.046 < 0.05) and anger (*a*3*b*3 = 0.034, *p* = 0.014 < 0.05). Since the direct effect of perceived threat on behavioral motivation was also significant (*t* = 2.97, *p* = 0.003 < 0.05, *c* = 0.151), we could conclude the mediator role for the emotions on the relationship between perceived threat and behavior motivation. Therefore, our results indicate that a high-threat, high-efficacy fear appeal has an impact on the perceived threat of e-cigarettes experienced by young people; this can then increase their emotions of fear and anger, as well as their motivation to avoid the risks associated with e-cigarettes.

Regarding perceived efficacy, although it was found to have a positive effect on the behavior motivation of young people (*t* = 3.41, *p* = 0.001 < 0.05, β = 0.169), none of the four groups of fear appeals could significantly increase their perceived efficacy. The above-mentioned findings provide an answer to research question Q1.

#### Fear Is Most Affected by Fear Appeals and Anxiety Is the Most Successful Emotion for Improving the Protection Motivation

The three models with fear, anxiety, and anger as the dependent variables had an *R*^2^ of 0.1157, 0.0759, and 0.0639, respectively. Thus, the fear model had the best effect, and perceived threat had the greatest effect on fear. This indicates that high-threat, efficient fear appeals have the greatest impact on fear, followed by anxiety and anger.

The β values obtained for the effect of fear, anxiety, and anger on behavioral motivation indicate that anxiety has the greatest impact on behavioral motivation, followed by anger and fear, respectively. This finding points out that low-certainty anxiety is more likely to influence young people’s avoidance behavior than high-certainty anger in regard to e-cigarettes. However, low-certainty fear has less impact on the risk avoidance of e-cigarette than anger. Overall, it can be seen that young people’s behavioral motivation is not determined by the deterministic appraisal tendency of emotion. These findings provide an answer to research question Q2.

### Youths, Young Women and Non-smokers Provide More Positive Feedback on Fear Appeals

#### Fear Appeals Have No Effect on Young People Aged 15–24, but Have a Significant Effect on Young People Aged 25–35

In this study, we focused on young people aged 35 and below. Based on the age, this cohort has been divided into two groups for analysis: 15–24 years and 25–35 years (the group under 15 years was extremely small and has thus been ignored).

The sample group aged 15–24 years (*n* = 196) did not respond in a significant way to any of the fear appeals. This might be due to the generally low cognitive level and risk recognition ability of this age group. Besides, they tend to have a natural curiosity about new things and make more independent judgments. Therefore, they may be slightly resistant to the persuasive content of fear appeals (Modecki, 2016). However, in the final behavioral motivation model, the perceived threat, perceived efficacy, anxiety, and anger of this group showed a significant positive effect on their behavioral motivation. This indicates that in future work regarding tobacco control, this group can be motivated to avoid e-cigarettes if appropriate material can be used to improve their perceived threat and perceived efficacy of the risks associated with e-cigarettes or to raise their anxiety and anger level.

In the sample group aged 25–35 years (*n* = 135), the same high-threat and efficient fear appeals could significantly increase the participants’ perceived threat (*p* = 0.030 < 0.05, β = 0.232), then increase their anger levels (*p* = 0.002 < 0.01, β = 0.273), and finally promote the protection motivation (*p* = 0.001 < 0.01, β = 0.269, *R*^2^ = 0.3327). The results are shown in Supplementary Figure 3. This demonstrates that this age group is more likely to perceive the e-cigarette-associated threats, since anger can be stimulated, and protection motivation can be accordingly generated. This may be because this age group has a greater abundance of social experiences than the younger age group (15–24 years). Besides, most of them have entered the workplace, experiencing greater work-life pressures and struggling to pay attention to their own health (Zhang and Liu, 2019). As a result, the positive feedback provided by this age group to e-cigarette fear appeals is more obvious.

#### High Fear Appeals Improve the Perceived Efficacy and Protection Motivation of Young Women

The results of applying the same above-mentioned path analysis on the sex of the participants demonstrate that e-cigarette fear appeals have a different path of action between young men and young women.

According to our findings, fear appeals had no significant impact on young men (*n* = 233), regardless of their level of threat and efficacy. However, for young women (*n* = 100), fear appeals that are both high-threat and high-efficacy could significantly improve their perceived efficacy and, consequently, their protection motivation. Although anxiety and anger had a significant impact on the behavioral motivation of women, the perceived efficacy of this group was not sufficient to significantly stimulate the three negative emotions. The results are shown in Supplementary Figure 4. Therefore, improving the perceived efficacy of young women using e-cigarette fear appeals can increase their motivation for e-cigarette risk aversion. Their protection motivation could be further enhanced if anxiety or anger can be stimulated by perceived efficacy, and the risk communicative effect of these fear appeals could then be improved.

#### Non-smokers Have a Higher Perception of the Risks Associated With E-Cigarettes

Since our participants included 317 smokers and only 16 non-smokers, we performed the above-mentioned path analysis only on smokers. The results showed that none of the four e-cigarette fear appeals had a significant impact on these smokers. Hence, the use of fear appeals to communicate the risks associated with e-cigarettes is not effective in young smokers.

Next, we performed independent sample *t*-tests on smoking history variables and perceived threat, perceived efficacy, fear, anxiety, anger, and behavioral motivation variables in the different experimental groups. In the situations of low-threat, including the low-threat, high-efficacy group and the low-threat, low-efficacy group, no significant differences were observed in any of the variables between smokers and non-smokers. Differences appeared in the situations of high-threat. In the high-threat, high-efficacy group, the perceived threat of young smokers was lower than that of young non-smokers (Supplementary Table 3). In the high-threat, low-efficacy group, the level of fear generated by e-cigarette fear appeals in young smokers was significantly lower than the level of fear generated in young non-smokers, as shown in Supplementary Table 4. As a result, the data collected from these two experimental groups show that the perception of the risks associated with e-cigarettes is much lower among young smokers compared with young non-smokers. The above-mentioned findings provide an answer to research question Q3.

## Discussion and Conclusion

In this study, a network control experiment based on the EPPM and ATF was performed, and the variable of public emotion was introduced to the use of fear appeals, such that this emotion was divided into three dimensions: fear, anxiety, and anger. Furthermore, the reactions of young people to e-cigarette fear appeals of different threat and efficacy levels and the mediating effect of the three emotions were investigated.

In terms of the overall theoretical framework, our study partially verified the theoretical hypothesis of the EPPM. The path taken by the risk control process was also essentially verified. However, it is worth noting that due to the small sample size, this study mainly focused on the role of emotion in the fear appeal path, while the phenomenon of the fear control process was not considered in this paper. Therefore, to design the experiment materials, this study referred to the conclusion of previous studies and set the intensity of fear at the raising part of the “inverted *U*-shaped” curvilinear relationship between fear and persuasion (Janis, 1967). In future research, the scale of the experimental samples could be expanded, and experimental material could be further modified to investigate this model.

The experimental results demonstrate that high-threat, high-efficacy fear appeals can significantly increase young people’s perceived threat of e-cigarettes, trigger their fear and anger and stimulate their protection motivation. Our experiment shows that fear appeals have no significant effect on the young people’s perceived efficacy, regardless of their level of threat and efficacy. However, high-threat, high-efficacy (β = 0.134^∗^) and high-threat, low-efficacy (β = 0.141^∗^) fear appeals can significantly improve the perceived effectiveness of the recommendations (one of the subdivision dimension of perceived efficacy). Our study indicates that high-threat fear appeals still have a significant positive impact on perceived efficacy, which can effectively improve the public’s willingness to protect themselves. This may be because most of the current discussions and shared information about e-cigarettes are about the hazards of e-cigarettes, industry development and related control policies in China. However, the relevance between e-cigarettes on the one side and general public and effective methods of tobacco control on the other side is rarely considered. Chen Hong and Hao Xiqun have conducted research on tobacco control reports appearing in the People’s Daily. Their study included 165 reports, among which only 8 reports contained a description of the effectiveness of the behavior recommended by the disseminator, and only 6 reports include a description of the people’s ability to adopt the recommended behavior. Their results indicate that the effectiveness of publicity surrounding the control of tobacco products in China is too low for fear appeals. They also found that the same is true for the control of e-cigarettes in China. This can lead to low public awareness and recognition of the self-protection suggestions proffered by professionals (Hong and Xiqun, 2013). Therefore, various levels of e-cigarette fear appeals do not have a significant impact on young people’s perceived efficacy.

The results of this study also indicate that fear appeals have different effects on young people depending on the factors of age, sex and whether they are smokers. In terms of age, fear appeals have no effect on young people aged 15–24 years, regardless of their level of threat and efficacy. For young people ages 25–35 years, high fear appeals have a significant positive effect on their perceived threat, and these appeals significantly improve their anger levels and protection motivation. Therefore, high fear appeals are more effective for controlling the use of e-cigarettes in the older age category. In terms of sex, high fear appeals can effectively enhance the efficacy perception and thus protect motivation behavior of young women. In young men, fear appeals do not have a significant effect on the perception of threat or efficacy, regardless of their level. E-cigarette fear appeals are more effective when used with young women compared with young men. Regarding the smoking history, young smokers produce no significant responses to fear appeals, while young non-smokers have higher levels of perceived threat and fear than smokers. It can be observed that young non-smokers have stronger perceptions of the risks involved with e-cigarettes than young smokers.

As a result of the previous analysis, this study proposes three suggestions for the future e-cigarette prevention and control. Firstly, the public should not only be informed about the severity of e-cigarette risks but they should also be provided with specific and feasible risk aversion strategies. This will help to ensure that more young people are aware of the threats posed by e-cigarettes and motivate them to take corresponding measures. Secondly, instead of relying solely on data and scientific research to “preach” to the public, effective e-cigarette risk aversion methods that are closely related to the daily life of young people should be provided by the government and relevant institutions (Jiang and Gong, 2015). In order to encourage them to adopt corresponding protective behavior, tobacco control work should be relevant to their daily lives and enhance their understanding of tobacco control and sense of efficacy. Thirdly, to successfully communicate the risks associated with e-cigarettes to people of different age, sex and smoking history, the messages to be delivered need to be adaptable. For young people aged between 25 and 35 years, high-threat, high-efficacy fear appeals can be used to communicate risks, particularly the severity and susceptibility of e-cigarette risks. In the case of young men aged between 15 and 24 years and young smokers, a single fear appeal has no significant impact on them. However, we can still change their cognitive structure, improve their perception of threat and efficacy and enhance their protection motivation by improving their knowledge of both the risks associated with e-cigarettes and effective protective behavior. For young women, we can improve their perception of efficacy using high fear appeals. Their ability to avoid e-cigarette risks and promote their protective behavior can be enhanced by informing them about effective e-cigarette risk prevention methods. For young non-smokers with a strong perception of the risks posed by e-cigarettes, we can continue to both popularize scientific knowledge related to e-cigarette risks and enhance their perception of threat and efficacy, so that they will continue to maintain a healthy lifestyle and refrain from using e-cigarettes.

In future research, the theoretical study of e-cigarettes could be further explored. On the one hand, when fear appeals on the risks posed by e-cigarettes are studied, the correlation dimension could be the focus of further research instead of examining the susceptibility dimension of threat perception. The susceptibility dimension examined in this study involved asking the participants about the possibility of being harmed by e-cigarettes, and the responses were rated on a 7-point Likert scale. This expression is abstract, and the term “harmful effects of e-cigarettes” refers to the harmful effects on people’s bodies. However, the harm caused by e-cigarettes has broader negative effects, such as affecting family relations, causing social contradictions, creating economic pressures, etc. Therefore, future research should consider the correlation dimension of e-cigarette risks. Experimental participants could be asked to measure how they are correlated with the high-risk situation. If the degree of correlation is high, then the participants are more likely to be threatened by e-cigarettes, and the perceived threat in this dimension is high.

On the other hand, the impact of fear, anxiety, and anger on the public’s protection motivation could be further studied. Previous studies have shown that fear is a form of emotion with low certainty and control, and anxiety is similar to fear. However, anger is opposite in nature to both these emotions (Yang and Chu, 2018). Their results show that these three emotions can have a significant positive impact on behavioral motivation. Anxiety has the greatest impact on behavioral motivation, followed by anger and fear. Their study indicates that the appraisal of certainty and control in the case of e-cigarettes is not the core topic affecting the protection motivation. Therefore, it is worth investigating which core appraisal factors lead these three emotions to produce the same behavioral decisions.

Our study has some limitations. Firstly, the experiment requires a larger sample. Although the four experimental groups had approximately the same size, they all had few participants. This might have caused some causal relationships to be inaccurately analyzed due to the small amount of data obtained, and thus the accuracy of the hypothetical model and experimental results might have been impacted. Secondly, the fear appeals used in the experimental material should be further improved and revised. Specifically, the number of words in the material that was used in this experiment differed across the four groups, which might have had an impact on the participants. Therefore, the control variables require further improvement. Finally, the questionnaire settings of this study require further modification. The average time allowed to complete the questionnaire was approximately 5–6 min, which might be slightly too long. The participants could then easily become bored when completing the questionnaire, and they might answer the questions at will. Therefore, the design of the experimental questionnaire should be further modified.

## Data Availability Statement

The original contributions presented in the study are included in the article/Supplementary Material, further inquiries can be directed to the corresponding author.

## Ethics Statement

Ethical review and approval was not required for the study on human participants in accordance with the local legislation and institutional requirements. Written informed consent to participate in this study was provided by the participants’ legal guardian/next of kin.

## Author Contributions

FW initiated the research, proposed the theoretical framework, developed the idea of manuscript, and offered correction idea. MJ designed the experimental materials and questionnaire, collected and analyzed the data, and wrote the manuscript. CS supervised and corrected the draft of manuscript, and drafted the revised manuscript. All authors read and approved the final manuscript.

## Conflict of Interest

The authors declare that the research was conducted in the absence of any commercial or financial relationships that could be construed as a potential conflict of interest.

## Publisher’s Note

All claims expressed in this article are solely those of the authors and do not necessarily represent those of their affiliated organizations, or those of the publisher, the editors and the reviewers. Any product that may be evaluated in this article, or claim that may be made by its manufacturer, is not guaranteed or endorsed by the publisher.

## Supplementary Material

Supplementary Figure 1 Hypothetical Model.

Supplementary Figure 2 Hypothetical Model Test Results.

Supplementary Figure 3 Young people aged 25–35.

Supplementary Figure 4 Young women.

Supplementary Table 1 The four dimensions of fear appeals.

Supplementary Table 2 Data relating to the participants.

Supplementary Table 3 Smoker or not ^∗^ Perceived threat *t*-test.

Supplementary Table 4 Smoker or not ^∗^ Fear *t*-test.
