# Supplementary material for: How Can E-Cigarette Fear Appeals Improve the Perceived Threat, Fear, Anger, and Protection Motivation of Young People
Source: Front Psychol. 2021 Aug 30;12:676363. doi: 10.3389/fpsyg.2021.676363 (PMC8435607; doi:10.3389/fpsyg.2021.676363)
Supplement: Supplementary Figure 1 — Hypothetical Model. [file Data_Sheet_1.docx]

Supplementary Material

# Supplementary Figures and Tables

## Supplementary Figures

**Supplementary Figure 1.** Hypothetical Model

**Supplementary Figure 2.** Hypothetical Model Test Results

**Supplementary Figure 3.** Young people aged 25-35

**Supplementary Figure 4.** Young women

## Supplementary Tables

| Dimension | Reference Documents | Specific Content |
| --- | --- | --- |
| High-threat | Articles on People’s Daily Online and the Chinese Science Communication website | Some surveys pointed out that in most e-cigarettes on sale, the actual content of highly addictive nicotine is much higher than the content given on the label. Furthermore, the damage caused to the immune system by e-cigarettes is nearly 7 times that caused by ordinary cigarettes. E-cigarettes can cause cardiovascular and lung diseases, cancer and other diseases. The amount of toxic heavy metals released by second-hand smoke from e-cigarettes far exceeds the amount released by traditional cigarettes. Harmful substances are absorbed without you being aware. Some low-quality e-cigarettes can also explode. For example, in March 2018, an e-cigarette being smoked by an American middle school student exploded, breaking and crushing several of the student’s teeth and damaging his chin bones. (A photograph of the horrific injuries was included). |
| Low-threat | Articles on People’s Daily Online | A survey pointed out that the long-term use of e-cigarettes can be addictive, leading to cardiovascular and lung disease, cancer and damage to the immune system. E-cigarettes also produce ‘second-hand smoke’, which will affect people close by. |
| High-efficacy | Articles on People’s Daily Online and the Jianshu Community | To avoid the hazards of e-cigarettes, regular medical examinations and early screening of diseases need to be conducted. When smokers are around, you should move away from them. After smoking or being exposed to passive smoke, you should wash your clothes and clean yourself thoroughly. Try to go to public places where there are ‘no smoking’ signs. Many people have recently shared their experiences of stopping e-cigarettes in online communities. These experiences are painful and unbearable at the beginning, but with each passing day they begin to feel better and have more control over all aspects of themselves. |
| Low-efficacy | Articles from the Chinese Center for Disease Control and Prevention | The best way to avoid the harmful effects of e-cigarettes is to stop smoking and to promote public health education activities within society. The harmful effects of using e-cigarettes should also be promoted to young people and families. However, one study has shown that those people who use e-cigarettes to stop smoking tobacco will eventually find it difficult to stop using e-cigarettes, as they are also addictive. |

**Supplementary Table 1.** The four dimensions of fear appeals

|  | **Total** | **Male** | **Female** |
| --- | --- | --- | --- |
| H-threat & H-efficacy | 89 | 61 | 28 |
| H-threat & L-efficacy | 75 | 54 | 21 |
| L-threat & H-efficacy | 77 | 59 | 18 |
| L-threat & L-efficacy | 92 | 59 | 33 |
| Total | 333 | 233 | 100 |

**Supplementary Table 2.** Data relating to the participants

| H-threat &  H-efficacy | Have you smoked cigarettes?（Mean ± Standard deviation） | | t | p |
| --- | --- | --- | --- | --- |
|  | Smoked (N=84) | Never smoked (N=5) |  |  |
| Perceived threat | 8.119 ± 0.218 | 10.600 ± 0.933 | -2.693 | 0.008 |

**Supplementary Table 3.** Smoker or not * Perceived threat t-test

| H-threat &  L-efficacy | Have you smoked cigarettes?（Mean ± Standard deviation） | | t | p |
| --- | --- | --- | --- | --- |
|  | Smoked (N=72) | Never smoked (N=3) |  |  |
| fear | 4.375 ± 0.229 | 5.000 ± 0.000 | -2.725 | 0.008 |

**Supplementary Table 4.** Smoker or not * Fear t-test t
